# Age-Related Changes in Neuroinflammation and Epigenetic Regulation in Mouse Ischemic Stroke Model

**DOI:** 10.3390/brainsci15080810

**Published:** 2025-07-28

**Authors:** Mari Kondo, Hayato Tamura, Eri Segi-Nishida, Hiroshi Hasegawa

**Affiliations:** 1 Laboratory of Hygienic Sciences, Kobe Pharmaceutical University, 4-19-1 Motoyamakita-machi, Higashinada-ku, Kobe 6588558, Japan; 2 Department of Biological Science and Technology, Faculty of Advanced Engineering, Tokyo University of Science, 6-3-1 Niijuku, Katsushika-ku, Tokyo 1258585, Japan

**Keywords:** ischemic stroke, microglia, astrocyte, cytokines, TLR4, HDAC7

## Abstract

**Background/Objectives**: The incidence and prevalence of ischemic stroke, a leading cause of death and disability worldwide, are significantly higher in older adults than in younger individuals. Senescence induces a variety of biological changes that influence the pathogenesis of diseases such as ischemic stroke, thereby necessitating age-specific medical treatments. However, the molecular mechanisms underlying age-related differences in ischemic stroke progression remain poorly understood. **Methods**: We compared the histological and molecular features of ischemic stroke in a photothrombotic mouse model, focusing on 9-week-old (young) and 90-week-old (old) mice. **Results**: We found that microglial accumulation at the infarct region of the cerebral cortex was significantly lower in old mice than in young ones. This reduction in the microglial response was accompanied by a decrease in the morphological robustness of the astrocytes forming the glial scar. Furthermore, the mRNA expression of proinflammatory cytokines CXCL10, CCL2, and TNF-α, which were upregulated in the infarct region, was considerably higher in the old mice than in the young ones. Cytokine expression was well correlated with the mRNA levels of Toll-like receptor 4 (TLR4), a key regulator of neuroinflammation in old mice, but less correlated with them in young mice. Interestingly, *Tlr4* mRNA expression in young mice was negatively correlated with the mRNA expression of the epigenetic regulator HDAC7, whereas this correlation was positive in old mice. **Conclusions**: These findings suggest that age-dependent changes in epigenetic regulation, such as the interaction between HDAC7 and TLR4, may contribute to the distinct pathological progression of ischemic stroke in older individuals.

## 1. Introduction

Stroke is a cerebrovascular disease that leads to neurological impairment caused by localized damage to the central nervous system. It is the second leading cause of death worldwide, accounting for approximately 7 million deaths annually. Even when not fatal, stroke is the third-leading cause of adult disability, with half of the affected individuals experiencing significant impairments that hinder independent living and require long-term medical care. Given these factors, stroke imposes a substantial burden on healthcare systems worldwide. While stroke can occur at any age, its incidence and prevalence increase with age. Approximately 75% of strokes occur in individuals aged ≥65 years [1]. Globally, the number of elderly individuals (≥65 years) is expected to rise; consequently, the incidence of stroke among the elderly is also predicted to increase. This trend is anticipated to place an even greater burden on healthcare systems [2].

Stroke is classified into ischemic stroke and hemorrhagic stroke, with the former accounting for approximately 85% of all cases globally [3,4,5,6]. Ischemic stroke is caused by the blockage of cerebral blood flow due to a thrombus, leading to a localized deficiency of oxygen and nutrients in the infarct area, resulting in irreversible damage to the brain tissue (infarct core) [5,6,7]. Ischemia-induced ATP depletion disrupts ion concentration gradients, causing cellular depolarization, calcium influx, and excessive release of glutamate neurotransmitters, ultimately resulting in neuronal and glial cell necrosis in the infarct core region [5,6,8]. The damaged cells in the infarct core and surrounding penumbra region, along with their debris, induce noninfectious inflammation, expanding the injured area. The inflammatory response involves various cell types, such as microglia, astrocytes, monocytes, and neutrophils, which produce proinflammatory cytokines. These cells contribute to the promotion of brain inflammation by clearing the necrotic tissue after cerebral ischemia. Properly controlled inflammation is crucial for tissue repair in the infarct region [9,10].

The incidence of ischemic stroke is higher in the elderly, and the outcomes of the condition vary with age. Moreover, age is a determinant of medical treatment efficacy [11]. Therefore, the mechanical differences in ischemic injury between younger and older patients should be understood, and tailored treatments should be developed [11]. However, many basic studies on stroke, including our own, have utilized young animal models. A few previous studies on ischemic stroke conducted using old mice have revealed that the inflammatory and immune responses are more sever in them [12,13]. In this study, we compared the pathological differences between young mice and old ones using a photothrombotic mouse model, with a particular focus on glial cells and the regulatory mechanisms of inflammation.

## 2. Materials and Methods

### 2.1. Animals

Male young (9-week-old) and old (90-week-old) ICR mice were purchased from Japan SLC, Inc. (Shizuoka, Japan). They were raised in the animal facility at Kobe Pharmaceutical University and acclimatized in individual housing for at least one week before the onset of the experiment. They were randomly assigned to histological and gene expression analyses. All procedures of the animal experiments in this study were conducted following the Guidelines for Proper Conduct of Animal Experiments of the Science Council of Japan.

### 2.2. Photothrombotic Ischemic Stroke Model

The photothrombotic ischemic stroke model was induced based on previous methods, with minor modifications [14]. Briefly, the mice were anesthetized with 0.3 mg/kg body weight (BW) of medetomidine hydrochloride, 4 mg/kg BW of midazolam, and 5 mg/kg BW of butorphanol to achieve the desired level of anesthesia. The scalps of the mice were shaved, and 10 mm square incisions were made to expose their skulls. Then, 10 mg/mL rose bengal (Waldeck GmbH and Co KG., Münster, Germany) dissolved in saline was injected intravenously through the jugular vein at 5 μL/g BW. After five minutes, the skull’s surface was dried by wiping with 70% ethanol. The somatosensory cortical area of the brain was then illuminated with a 50 mW laser at 532 nm for four minutes to induce blood coagulation. Thereafter, the scalps were sutured, and 75 μg/kg BW of atipamezole (an antagonist of medetomidine) was administered for quick recovery from anesthesia. The mice were returned to their home cage and shielded from light for 48 h, using a foil.

### 2.3. Histological Analyses

The mice were deeply anesthetized and transcardially perfused with 4% paraformaldehyde in phosphate-buffered saline (PBS). The collected brain was fixed in 4% paraformaldehyde in PBS at 4 °C for five hours and cryoprotected in 30% sucrose in PBS at 4 °C overnight. The brain was embedded in the O.C.T. compound (Sakura Finetek Japan Co. Ltd., Tokyo, Japan). Then, 30 μm tissue sections were prepared with a cryostat (SLEE medical GmbH, Mainz, Germany).

Immunohistochemistry was performed as described in our previous manuscript with slight modifications [15]. The tissue sections were fixed in 4% PFA in PBS for five minutes, briefly washed with PBS thrice, and permeabilized in 0.5% Triton X-100 in PBS for 10 min at room temperature. Thereafter, the sections were washed thrice in PBS and incubated in citrate buffer (pH = 6.0) at 75 °C for 40 min for antigen retrieval. Thereafter, they were then washed thrice in PBS and blocked in 1.5% fetal bovine serum in PBS for one hour at room temperature. After the blocking step, they were incubated with the following primary antibodies at 4 °C overnight: anti-ionized calcium-binding adaptor molecule 1 (IBA1)/allograft inflammatory factor 1 (AIF1) (019-19741, Fujifilm Wako Pure Chemical Co., Ltd., Osaka, Japan) and glial fibrillary acidic protein (GFAP) (Z0334, DAKO/Agilent, Santa Clara, CA, USA). After washing with PBS thrice, they were incubated with Alexa488-conjugated anti-rabbit IgG (711-545-152, Jackson ImmunoResearch, West Grove, PA, USA), together with 4′,6-diamidino-2-phenylindole, in 1.5% fetal bovine serum in PBS, at room temperature for two hours. After washing thrice with PBS, they were mounted with Fluoromount-G (SouthernBiotech, Birmingham, AL, USA).

All images were acquired using a BZ-X810 microscope (KEYENCE, Osaka, Japan). Processing and quantification were performed using Fiji (ver. 2.16.0), an open-source software for image manipulation.

For the quantification of GFAP-positive signals, the fluorescent intensity in the areas within 200 μm from the boundary between the core and penumbra region was obtained on Fiji and the background signals in the contralateral region with almost no GFAP-positive signals were subtracted.

### 2.4. RNA Purification and RT-qPCR

RNA extraction and quantitative reverse transcription polymerase chain reaction (RT-qPCR) analysis were performed as described in previous manuscript [16]. The mice were deeply anesthetized and transcardially perfused with PBS. The ipsilateral and contralateral sides of the cerebral cortex were collected, including the infarct core for the ipsilateral side. The collected tissues were stored in Sepasol-RNA I super G solution (Nacalai Tesque, Inc., Kyoto, Japan) at −80 °C, followed by homogenization and total RNA purification as per the manufacturer’s instruction. The concentration of the purified RNA was determined using a NanoDrop 1000 spectrophotometer (Thermo Fisher Scientific, Wilmington, DE, USA). Complementary DNA was synthesized using ReverTra Ace reagent (Toyobo Co. Ltd., Osaka, Japan) according to the manufacturer’s instructions. The expression levels of the target genes were determined using the CFX Connect real-time PCR detection system (Bio-Rad Laboratories Inc., Hercules, CA, USA), where PCR amplification was performed using SsoAdvanced Universal SYBR Green Supermix (Bio-Rad Laboratories) with the primer pairs listed in Supplementary Table S1. The PCR parameters were as follows: one minute of initial DNA polymerase activation, DNA denaturation at 95 °C, and 40 cycles of denaturation at 95 °C for 15 s and primer annealing-fragment extension at 60 °C for 30 s. The melting curves of the real-time PCR products were analyzed from 65 °C to 90 °C. Differences in gene expression, expressed as fold change, were calculated using the ΔΔCt method with Microsoft Excel (Microsoft, Co., Redmond, WA, USA). *Rplp2* was used as a reference gene for normalizing the expression.

### 2.5. Statistical Analyses

Results are presented as the mean ± standard deviation. Mean values were compared between two groups using Student’s *t*-test and among four groups using the two-way factorial analysis of variance (ANOVA), followed by the Tukey–Kramer post hoc test, as indicated in the figure legends. RT-qPCR data were mathematically analyzed for multivariate or correlation analyses with R software (ver. 4.5.1). Pearson’s correlation coefficient was calculated using the R software to examine the relationship between the two factors.

## 3. Results

### 3.1. Different Astrocyte Morphologies in the Penumbra Region Between Young and Old Mice

**Figure 1:**
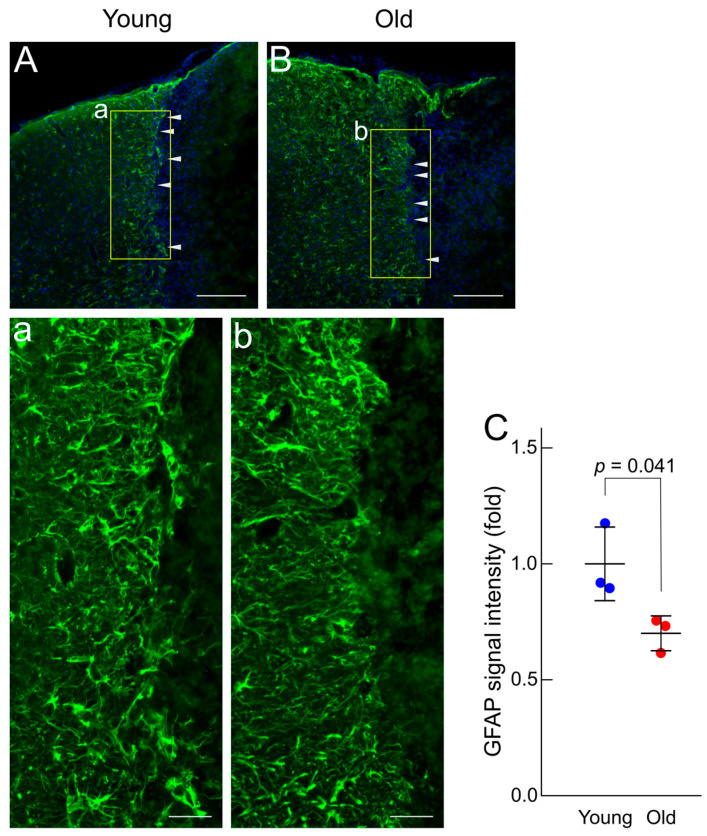
Impaired formation of the glial scar by reactive astrocytes in old mice. (**A**,**B**) Cerebral cortex sections from the ipsilateral side of young and old mice, seven days after photothrombosis, were immunostained with the anti-GFAP antibody (*n* = 3). The yellow square regions in (**A**) and (**B**) are shown at higher magnification in (**a**) and (**b**), respectively. The white arrowheads indicate the boundary between the ischemic core and penumbra regions. Scale bars: 200 μm (**A**,**B**), 50 μm (**a**,**b**). (**C**) The intensity of the GFAP signal was quantified as described in the Section 2 of the manuscript. Values are expressed relative to the average in young mice. Statistical significance was determined using Student’s *t*-test.

We first examined the accumulation and reactivity of astrocytes in the penumbra region of young and old mice seven days after ischemic stroke, when the astrocytic glial scar begins to form (Figure 1; Supplementary Figure S1). Immunostaining with the anti-GFAP antibody indicated that GFAP-positive astrocytes were strictly excluded from the core region and lined up along the boundary between the core and penumbra regions in the young mice. These astrocytes exhibited thick processes with a strong GFAP signal and formed a clear boundary between the core and penumbra regions. In old mice, astrocytes also accumulated at the boundary between the core and penumbra regions; however, they did not form an apparent boundary, and their processes—filamentous and thinner than those in young mice—extended toward the core region. In contrast, GFAP-positive astrocytes were observed farther from the boundary in the old mice; however, fewer of them were present in the young mice. Thus, reactive astrocytes accumulate more effectively toward the core region in young mice than in old ones.

### 3.2. Noninfectious Inflammatory Reaction in the Early Phase with Different Characteristics in Young and Old Mice

**Figure 2:**
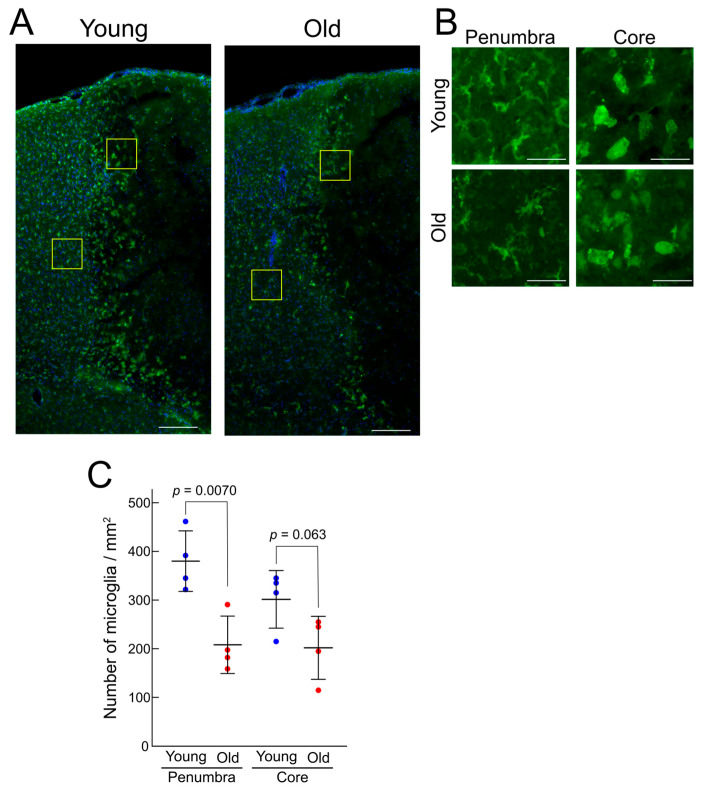
Reduced microglial accumulation in the infarct region in old mice. Cerebral cortex sections from the ipsilateral side of young and old mice, three days after photothrombosis, were immunostained with an anti-IBA1/AIF1 antibody (*n* = 4). (**A**,**B**) Representative images. The yellow square regions in (**A**) are shown at higher magnification in (**B**). Scale bars: 200 μm (**A**), 50 μm (**B**). (**C**) Microglia in the penumbra and ischemic core regions within 200 μm from the boundary between these regions were counted and normalized to the area. Statistical significance was determined using Student’s *t*-test.

In our previous studies, we indicated that astrocyte reactivity is regulated by microglial activity and that intercellular communication between astrocytes and microglia is critical for ischemic stroke pathogenesis and progression [14]. Therefore, we hypothesized that microglial activity in the acute phase of ischemic stroke might differ between young mice and old ones. Microglial activity in the ischemic region of the cerebral cortex was examined by immunostaining with an anti-IBA1/AIF1 antibody, which stains activated microglia, three days after photothrombosis, when post-ischemic microglial activation and inflammation peaks (Supplementary Figure S1). Microglial activation, indicated by the IBA1/AIF1-positive signals, was observed in the infarct regions of young and old mice (Figure 2A). As previously reported, microglial morphology differed between the core and penumbra regions—those in the core region were amoeboid, and those in the penumbra region were ramified. There was no apparent difference in the microglial morphology and IBA1/AIF1 signal intensity between young mice and old ones (Figure 2B). However, the number of IBA1/AIF1-positive microglia in the core and penumbra regions was lower in the old mice than in the young ones (Figure 2C). These findings suggest that microglial accumulation in the early phase after ischemic stroke, as well as astrocyte reactivity in the late phase, are both weaker in old mice than in young ones.

**Figure 3:**
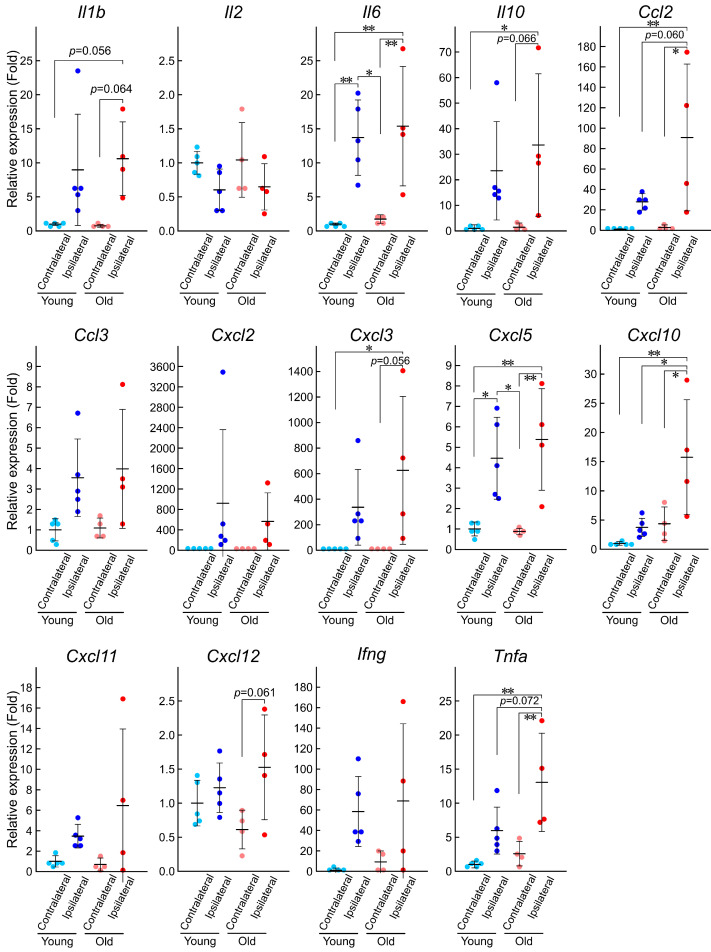
mRNA expression of inflammatory cytokines in the infarct region of young and old mice. The expression levels of inflammatory cytokines three days after photothrombosis were measured by RT-qPCR as described in the Materials and Methods section of the manuscript (young, *n* = 5; old, *n* = 4). Values are expressed relative to the average of the contralateral side in young mice. Statistical significance was determined using the Tukey–Kramer post hoc test. * *p* < 0.05, ** *p* < 0.01.

To investigate whether inflammatory cytokine production differs between young mice and old ones, we examined the mRNA expression of proinflammatory and anti-inflammatory cytokines by RT-qPCR (Figure 3 and Table 1). All cytokines examined here were upregulated or downregulated in the ipsilateral region compared with the contralateral region three days after photothrombosis, as revealed by the two-way factorial ANOVA (Table 1). However, only the mRNA expression of *Cxcl10*, *Ccl2*, and *Tnfa* was specifically affected by aging. An interaction effect of ischemic stroke and aging was observed for *Cxcl10* and *Ccl2*, with *p*-values of 0.079 and 0.075, respectively, but not for *Tnfa*. The post hoc analysis performed using the Tukey–Kramer test revealed that the mRNA expression of *Cxcl10* in the ipsilateral region was significantly increased with aging (Figure 3). The expression levels of *Ccl2* and *Tnfa* in the ipsilateral region also increased significantly with aging, with *p*-values of 0.060 and 0.072, respectively (Figure 3). *Cxcl12* expression was uniquely affected in old mice (where it was induced in the ipsilateral side compared to the contralateral side) but not in young ones. These results indicate that many inflammatory cytokines are induced by the ischemic lesion in the cerebral cortex and that the induction of some specific cytokines is age-dependent, which may be involved in the pathological differences observed in ischemic stroke depending on age.

### 3.3. Age-Dependent Variation in TLR—Cytokine Correlation After Ischemic Stroke

As the expression levels of *Cxcl10*, *Ccl2*, and *Tnfa* mRNAs are age-dependent, we sought to identify the age-dependent regulatory mechanism underlying the expression levels of these mRNAs. TLR4 is known as a master regulator of inflammatory cytokines [17,18] and acts as a receptor for DAMPs, which are released during cell death in ischemic stroke [19]. Recent studies suggested that TLR4 exacerbates stroke prognosis in a rodent stroke model with middle cerebral artery occlusion (MCAO) [20,21]. To determine whether TLR4 is involved in the age-dependent differences in cytokine induction in the infarct region, the mRNA expression of *Tlr4* was examined by RT-qPCR analysis.

**Figure 4:**
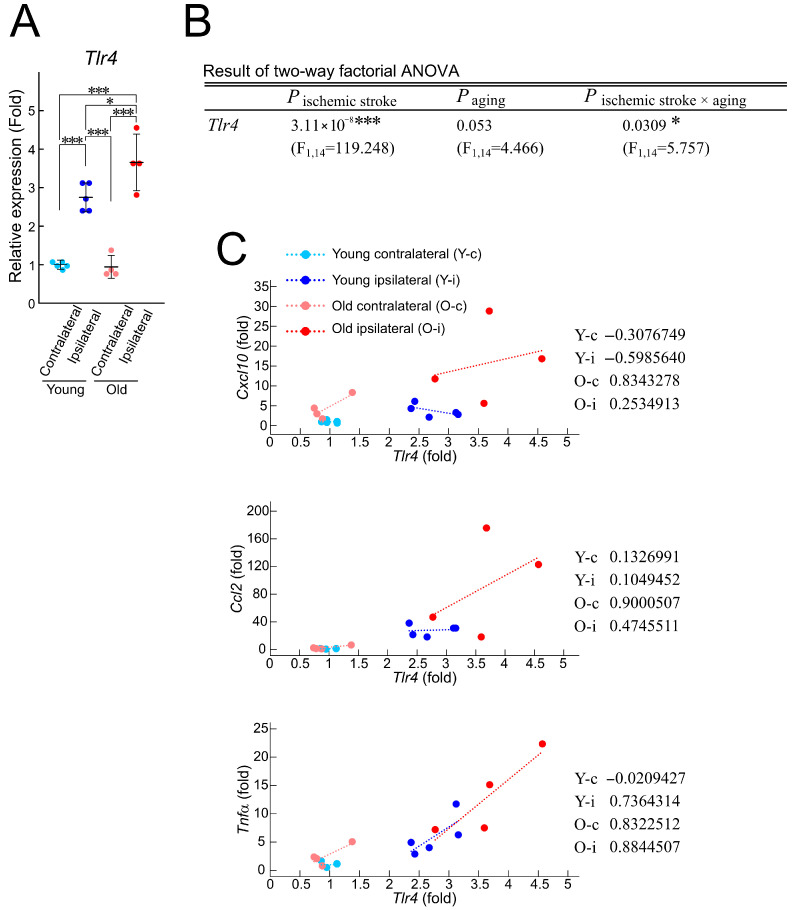
Relationship between the mRNA expression of inflammatory cytokines and *Tlr4*. (**A**) mRNA expression of *Tlr4* in the contralateral and ipsilateral (infarct) regions of young (*n* = 5) and old (*n* = 4) mice three days after photothrombosis. Values are expressed relative to the average of the contralateral side in young mice. Statistical significance was determined using the Tukey–Kramer post hoc test. (**B**) The effects of ischemic stroke, aging, and their interaction on *Tlr4* mRNA expression were analyzed using the two-way factorial ANOVA. * *p* < 0.05, *** *p* < 0.001. (**C**) Results of the correlation analysis between inflammatory cytokines and *Tlr4*. The correlation coefficients for each condition are displayed.

*Tlr4* expression was significantly upregulated in the ipsilateral region compared to the contralateral region in both young and old mice, with higher induction observed in old mice than in young ones (Figure 4A). The two-way factorial ANOVA analysis revealed that ischemic stroke significantly affected *Tlr4* expression (Figure 4B). In addition, *Tlr4* expression was influenced by aging and by an interaction between ischemic stroke and aging. We further investigated whether there was a correlation between *Tlr4* mRNA expression and the expression of *Cxcl10*, *Ccl2*, and *Tnfa* mRNAs (Figure 4C). In young mice, the expression levels of *Cxcl10* and *Tlr4* mRNA showed a negative correlation in the ipsilateral and contralateral regions. In contrast, this correlation became a strong positive one in both regions in old mice. There was no significant correlation in the expression levels of *Ccl2* and *Tlr4* mRNAs in the young mice, but there was a significant positive correlation between them in the old mice in both the ipsilateral and contralateral regions. Thus, the expression levels of CXCL10 and CCL2 appear to be associated with TLR4 signaling in old mice but not young ones. For *Tnfa*, no significant correlation with *Tlr4* was observed in the contralateral region of the young mice (Figure 4C), whereas a strong positive correlation emerged in the ipsilateral region. In the old mice, strong positive correlations were observed in the ipsilateral and contralateral regions. These results suggest that aging increases the basal expression of TNF-α. Taken together, these findings suggest that the ischemic stroke-induced expression of certain cytokines is influenced by age-dependent changes in their relationship with TLR4.

### 3.4. HDAC7 as an Age-Dependent Regulator Through Epigenetic Modification

**Figure 5:**
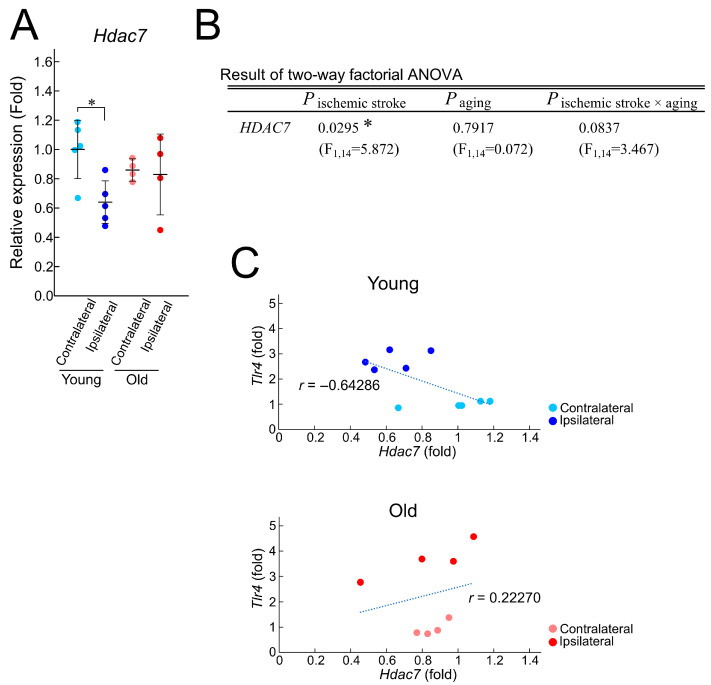
Relationship between the mRNA expression of *Hdac7* and *Tlr4*. (**A**) mRNA expression of *Hdac7* in the contralateral and ipsilateral (infarct) regions of young (*n* = 5) and old (*n* = 4) mice three days after photothrombosis. Values are expressed relative to the average of the contralateral side in young mice. Statistical significance was determined using the Tukey–Kramer post hoc test. (**B**) The effects of ischemic stroke, aging, and their interaction on *Hdac7* mRNA expression were analyzed using the two-way factorial ANOVA. * *p* < 0.05. (**C**) Results of correlation analysis between *Tlr4* and *Hdac7*. The correlation coefficients for each condition are displayed.

Epigenetic modification is a well-known molecular mechanism involved in age-related biological changes [22,23]. To investigate its potential involvement in the age-dependent cytokine induction that occurs in ischemic regions, we assessed the expression of histone deacetylases (HDACs), a representative family of enzymes involved in epigenetic modification [24]. HDAC inhibitors have been shown to suppress TLR-induced inflammatory responses, suggesting the functional roles of HDACs in inflammation [25]. Among the 11 members of the HDAC family, we focused on HDAC7, because it has been identified as a critical regulator of TLR4-mediated inflammatory responses in macrophages [26]. HDAC7 has been found to increase the astrocytic expression of inflammatory cytokines [27], supporting the hypothesis that HDAC7 is involved in stroke progression. To determine whether *Hdac7* mRNA expression is affected by aging, we performed the RT-qPCR analysis. In young mice, *Hdac7* mRNA expression was significantly decreased in the ipsilateral region of the cerebral cortex compared to the contralateral one. In contrast, in old mice, *Hdac7* expression was similar between the two regions (Figure 5A). The two-way factorial ANOVA analysis showed that *Hdac7* mRNA expression was affected by ischemic stroke and tended to be influenced by the interaction between ischemic stroke and aging (Figure 5B). To assess whether age-related changes in *Hdac7* expression contribute to TLR4-associated alterations in *Cxcl10* and *Ccl2* mRNA expression in young and old mice, we analyzed the correlation between the expression levels of *Hdac7* and *Tlr4*. Our results indicated a negative correlation between *Hdac7* and *Tlr4* mRNA in young mice, whereas it was a positive correlation in old mice (Figure 5C). These findings indicate that aging alters not only the expression pattern of *Hdac7* mRNA but also its association with *Tlr4*, suggesting a potential role for HDAC7 in the age-dependent regulation of inflammatory responses following ischemic stroke.

## 4. Discussion

A hallmark of the pathological mechanism of ischemic stroke is cell death and inflammation caused by ischemia due to thrombosis. The most common treatment for ischemic stroke in humans is early reperfusion therapy—either through intravenous thrombolysis using a tissue plasminogen activator or mechanical thrombectomy—to restore blood flow and salvage the penumbral region [9,28]. Although early reperfusion is crucial, it does not address the ensuring inflammation. Ischemia–reperfusion can lead to the production of reactive oxygen species and glutamate excitotoxicity, which may further exacerbate inflammation and expand tissue damage. Therefore, a treatment strategy that includes not only rapid reperfusion but also inflammation control is necessary. However, to the best of our knowledge, no therapy specifically targeting poststroke inflammation [4,29,30]. In recent years, therapeutic strategies aimed at modulating inflammation have gained increasing attention [31,32].

**Figure 6:**
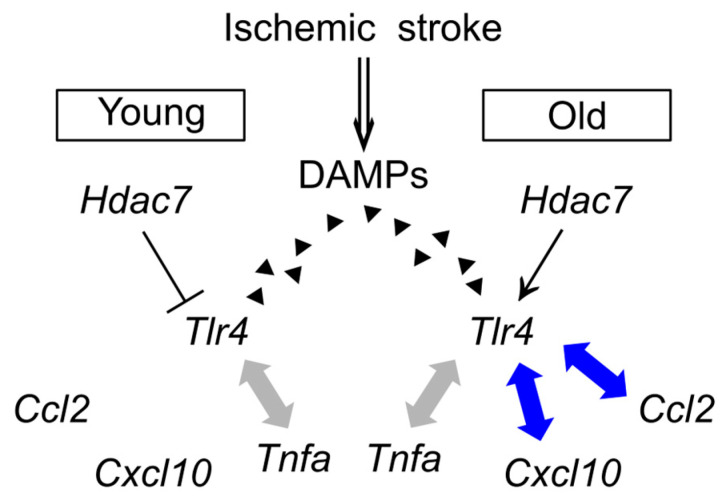
Schematic summary of this study. The gray and blue arrows represent the associations between *Tlr4* mRNA expression and inflammatory cytokine expression.

In this study, we demonstrated that among the inflammatory cytokines upregulated in the infarct region of the mouse photothrombotic ischemic stroke model, the induction levels of *Cxcl10*, *Ccl2*, and *Tnfa* mRNAs were substantially higher in the old mice than in the young ones. Moreover, the expression levels of these cytokines were associated with the *Tlr4* mRNA level. Notably, the expression level of *Tlr4* mRNA was negatively correlated with the *Hdac7* mRNA level in the young mice, whereas this correlation was either absent or reversed in the old mice. These findings suggest that the relationship between *Hdac7* and *Tlr4* expression levels is age-dependent and that age-related changes in this interaction may contribute to the differential induction of specific inflammatory cytokines in the infarct region, ultimately influencing the pathological outcomes in young and old mice (Figure 6).

### 4.1. Differences in Glial Cells in the Infarct Region Between Young Mice and Old Ones

Following ischemic injury, microglia are rapidly activated and accumulate in the infarct region during the acute phase, followed by the delayed increase in astrocyte reactivity [14]. Reactive astrocytes extend the elongated processes surrounding the ischemic lesion and form a glial scar within a few days post-injury [31,33]. This glial scar serves to isolate the ischemic core from the penumbra region, thereby preventing the diffusion of harmful factors—such as DAMPs—from the core into the surrounding penumbra and distant healthy areas. It also contributes to the production of neuroprotective factors [31,33,34]. Despite its positive functions, the glial scar can also have detrimental effects on neural repair by forming a physical barrier and secreting molecules that inhibit axonal growth [34]. In addition, reactive astrocytes have been reported to protect preexisting blood vessels within the ischemic core and to facilitate vascular repair and remodeling [33].

In this study, we observed that GFAP-positive astrocytes in old mice exhibited thinner processes than those in young mice, with their processes extending toward the infarct core region at seven days after photothrombotic stroke (Figure 1). This morphological change suggests that glial scar formation is weaker in older mice than in younger ones. Impaired glial scar formation in old mice may result in the leakage of DAMPs from the ischemic core into the surrounding regions and reduced production of neuroprotective factors, culminating in further neuronal and glial cell damage. Thus, age-related differences in glial scar formation may contribute to the poorer ischemic stroke outcome observed in the elderly. Conversely, astrocytes play a role in the development of edema after ischemic stroke onset through aquaporin (AQP4)-mediated water transport [35]. It has been reported that the degree of edema is milder in the elderly than in the young [36,37]. Interestingly, AQP4 controls astrocytic processes and motility, and the expression and localization of AQP4 have both been shown to change with age [38,39]; therefore, the difference in astrocytic shape between young and old mice may be related to the degree of edema. Future studies should investigate the long-term pathological consequences in both young and old mice to further elucidate the impact of glial remodeling on stroke recovery.

### 4.2. Noninfectious Neuroinflammation and Specific Inflammatory Cytokine Upregulation in Old Mice

Increase in astrocyte reactivity is reportedly influenced by microglial activation [40,41]. Indeed, our previous study demonstrated that the inhibition of microglial activity by minocycline enhanced astrocyte reactivity [14]. Because astrocytic reactivity was weaker in the old mice than in the young ones, we initially hypothesized that microglial activity would be more pronounced in the former than in the latter. However, contrary to our expectation, the microglial counts in the infarct core and penumbra regions were significantly lower in the old mice than in the young ones three days after photothrombosis (Figure 2). The signal intensity of IBA1/AIF1, a marker of microglial activity, did not show any obvious difference between age groups. These results suggest that although microglial activity may be maintained in old mice, microglial recruitment to the infarct region could be impaired, possibly due to diminished neuron–microglia interactions and/or increased sensitivity to migration-inhibitory signals [42,43]. Interestingly, despite the microglial count reduction, the expression of most inflammatory cytokine was not decreased in old mice; rather, some cytokines were upregulated in the infarct region (Figure 3).

Inflammatory cytokines play crucial roles in the onset and progression of ischemic stroke [8,44,45,46]. Consistent with previous reports, all inflammatory cytokines examined here were upregulated in the infarct region after ischemic stroke (Figure 3). Several studies have suggested that cytokine expression is generally increased in an older brain under both healthy and pathological conditions [47,48,49]. In our photothromobotic ischemic stroke model, the upregulation of *Cxcl10* and *Ccl2* was particularly pronounced in the old mice (Figure 3). While previous studies have reported the induction of *Cxcl10* and *Ccl2* in stroke, the age-dependent enhancement of their induction is a novel finding of our study.

Induced expression of both CXCL10 and CCL2 has also been observed in human cases of ischemic and hemorrhagic stroke [50,51]. CXCL10, also known as interferon-γ-inducible protein 10 (IP-10), is a chemokine secreted by multiple CNS cell types—including endothelial cells, neurons, microglia, and astrocytes—in response to interferon-γ, TNF, and various Toll-like receptor ligands [52,53,54]. It has also been implicated in neuroprotection through vascular pruning, extracellular matrix remodeling, and apoptosis inhibition by binding its receptor CXCR3 [55,56,57]. While CXCL10 is induced in the infarct region after stroke (which is consistent with our findings), its suppression has been shown to improve synaptic function and sensorimotor outcomes in the photothrombotic stroke model [58]. CCL2 facilitates immune cell migration to the injury sites by binding to its receptor CCR2 [59,60]. Mice deficient in either *Ccl2* or *Ccr2* exhibit impaired macrophage recruitment during inflammation [61,62]. Beyond its chemotactic properties, CCL2 also increases the permeability of the blood–brain barrier [63,64,65]. Clinical studies have reported a correlation between CCL2 expression levels and poststroke outcomes, highlighting its relevance in human stroke pathology [51]. Given that *Cxcl10* and *Ccl2* have both been associated with stroke prognosis in humans, their increased expression in old mice compared with that in young mice is noteworthy (Figure 3) [66,67].

In this study, we did not examine the expression of the receptors for these cytokines. Because the biological effects of CXCL10 and CCL2 depend on the presence and distribution of their corresponding receptors, it is possible that aging alters the receptor expression levels or patterns. Differences in the receptor-expressing cell populations between young and old individuals may also affect cytokine signaling outcomes. Future studies should be conducted to investigate the receptor expression profiles and cellular localization in the context of aging.

### 4.3. In Old Mice, Cytokines with Upregulated Expression Exhibited an Altered Correlation with Their Master Regulator

TLR4 is a key regulator of cytokines [68,69]. Based on this, we examined the correlation between *Cxcl10*, *Ccl2*, and *Tlr4*, whose expression levels were elevated in the old mice. Per our findings, the patterns of correlation differed between the young mice and the old ones. Considering that the *Tlr4* gene is susceptible to epigenetic regulation through histone modification [70,71], one possible explanation for these differences could be the different epigenetic modifications of the *Tlr4* gene (which affect its induction in the ipsilateral region) between young and old mice. HDACs are major enzymes that mediate epigenetic transcriptional regulation by modifying histones [72]. Among the members of HDAC family, HDAC7 has been identified as a critical regulator of TLR4-mediated inflammatory responses in macrophages [26].

In young mice, *Hdac7* mRNA expression was significantly decreased in the ipsilateral region following photothrombotic stroke, and its expression showed a strong negative correlation with *Tlr4* (Figure 5). In contrast, in old mice, *Hdac7* mRNA levels did not significantly decrease in the ipsilateral region, and no correlation or a weakly positive correlation with *Tlr4* was observed. These findings suggest that TLR4-medated inflammation may be regulated by HDAC7 to suppress excessive inflammatory responses, and this regulatory mechanism appears to be impaired with aging. HDAC7 is considered unique among HDACs due to its relatively weak histone deacetylase activity and diverse molecular functions. It has been implicated in the regulation of various biological processes that occur in immune cells, osteoclasts, muscle cells, endothelial cells, and epithelial cells [25,73,74]. Although the in vivo role of HDAC7 in the TLR pathway is not fully understood, previous studies have identified HDAC7 as a therapeutic target in immune-related diseases [25]. Given the observed changes in its expression and correlation with *Tlr4*, it is likely that age-related epigenetic mechanisms contribute to the altered regulation of inflammatory responses in stroke. Considering the limitation of this study, including the relatively small sample size and the unavailability of direct evidence at the protein-level analysis, future investigations on the roles of HDAC7 and other HDAC family members are required to further elucidate these mechanisms.

### 4.4. Future Perspectives

Aging, a phenomenon accompanied by a lot of changes in body properties, is characterized by systemic chronic inflammation with higher levels of inflammatory markers, referred to as inflammaging [75]. It has been demonstrated that systemic inflammation predisposes to ischemic stroke, whereas the involvement of inflammaging in the progression of ischemic stroke has not been investigated [76]. Aging is also associated with changes in certain metabolic parameters, such as blood glucose levels, insulin sensitivity, and lipid signaling and storage, which can also affect the pathological progression of stroke [77]. The effects of age-associated systemic changes on stroke progression should be investigated in future studies. Pathological outcomes and age/sex differences are also important from a clinical view point, and they should be clarified to elucidate the whole picture of age-related differences in the pathological progression of ischemic stroke.

## 5. Conclusions

In summary, we demonstrated that aging-specific inflammation plays a critical role in injury progression following photothrombotic stroke. Moreover, we emphasize that understanding the mechanisms underlying age-related inflammatory changes is essential for developing effective therapeutic strategies for ischemia in the elderly. By examining the relationship between master regulators and downstream cytokines, we suggest age-dependent alterations in gene expression networks. These differences contribute to enhanced cytokine induction in older brains and highlight a fundamental shift in inflammatory regulation with aging. Basic research focused on aging-related mechanisms provides vital insight for the development of targeted therapies aimed at improving outcomes in elderly individuals with ischemic stroke.

## Figures and Tables

**Table 1:** Effect of ischemic stroke and aging on inflammatory cytokine expression analyzed using the two-way factorial ANOVA.

mRNA	*P*_ischemic stroke_	*P*_aging_	*P*_ischemic stroke__×__aging_
*Il1b*	0.00234 **	0.77720	0.69833
	(F_1,14_ = 13.748)	(F_1,14_ = 0.083)	(F_1,14_ = 0.157)
*Il2*	0.0315 *	0.7981	0.9987
	(F_1,14_ = 5.710)	(F_1,14_ = 0.068)	(F_1,14_ = 0.000)
*Il6*	7.32 × 10^-5^ ***	0.620	0.846
	(F_1,14_ = 30.662)	(F_1,14_ = 0.257)	(F_1,14_ = 0.039)
*Il10*	0.00398 **	0.51329	0.54898
	(F_1,14_ = 11.835)	(F_1,14_ = 0.450)	(F_1,14_ = 0.377)
*Cxcl2*	0.0676	0.6560	0.6492
	(F_1,14_ = 3.924)	(F_1,14_ = 0.207)	(F_1,14_ = 0.216)
*Cxcl3*	0.00689 **	0.34562	0.34308
	(F_1,14_ = 10.012)	(F_1,14_ = 0.953)	(F_1,14_ = 0.963)
*Cxcl5*	0.000121 ***	0.605907	0.499443
	(F_1,14_ = 27.643)	(F_1,14_ = 0.279)	(F_1,14_ = 0.481)
*Cxcl10*	0.01170 *	0.00464 **	0.07939
	(F_1,14_ = 8.396)	(F_1,14_ = 11.314)	(F_1,14_ = 3.579)
*Cxcl11*	0.034 *	0.438	0.345
	(F_1,14_ = 5.523)	(F_1,14_ = 0.638)	(F_1,14_ = 0.956)
*Cxcl12*	0.0287 *	0.8440	0.1391
	(F_1,14_ = 5.946)	(F_1,14_ = 0.040)	(F_1,14_ = 2.459)
*Ccl2*	0.00415 **	0.06155	0.07462
	(F_1,14_ = 11.693)	(F_1,14_ = 4.130)	(F_1,14_ = 3.711)
*Ccl3*	0.00502 **	0.75379	0.83629
	(F_1,14_ = 11.045)	(F_1,14_ = 0.102)	(F_1,14_ = 0.044)
*Ifng*	0.00765 **	0.62959	0.95696
	(F_1,14_ = 9.683)	(F_1,14_ = 0.243)	(F_1,14_ = 0.003)
*Tnfa*	0.00126 **	0.03496 *	0.16053
	(F_1,14_ = 16.169)	(F_1,14_ = 5.451)	(F_1,14_ = 2.196)

* *p* < 0.05, ** *p* < 0.01, *** *p* < 0.001.

## Data Availability

The original contributions presented in this study are included in the article/Supplementary Material. Further inquiries can be directed to the corresponding author.

## Supplementary Materials

The following supporting information can be downloaded at: https://www.mdpi.com/article/10.3390/brainsci15080810/s1, Figure S1: Experimental process diagram, Table S1: Primer sequences used for RT-qPCR [16,78,79].

## Abbreviations

The following abbreviations are used in this manuscript:

AIF1	Allograft inflammatory factor 1
ANOVA	Analysis of variance
AQP4	Aquaporin-4
BW	Body weight
GFAP	Glial fibrillary acidic protein
HDAC	Histone deacetylases
IBA1	Ionized calcium-binding adaptor molecule 1
MCAO	Middle cerebral artery occlusion
PBS	Phosphate-buffered saline
RT-qPCR	Quantitative reverse transcription polymerase chain reaction
TLR	Toll-like receptor
