# Supplementary material for: Age-Related Changes in Neuroinflammation and Epigenetic Regulation in Mouse Ischemic Stroke Model
Source: Brain Sci. 2025 Jul 28;15(8):810. doi: 10.3390/brainsci15080810 (PMC12384191; doi:10.3390/brainsci15080810)
Supplement: Supplementary file 1 [file brainsci-15-00810-s001.zip › brainsci-3740756-supplementary.pdf]

Supplementary Materials:

Age-Related Changes in Neuroinflammation and Epigenetic Regulation in Mouse Ischemic Stroke Model

Mari **Kondo**, Hayato **Tamura**, Eri **Segi-Nishida**, Hiroshi **Hasegawa**

Supplementary Figure S1

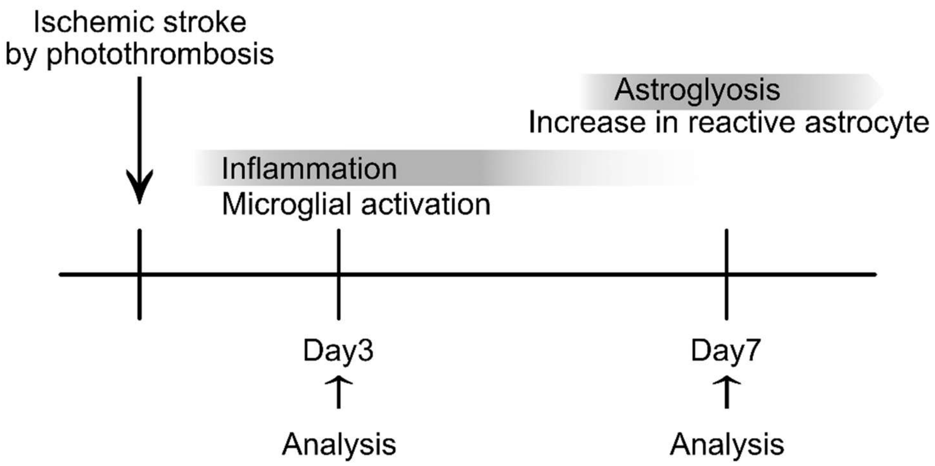

Figure S1. Experimental process diagram.

Supplementary Table S1

Primer sequences used for RT-qPCR.

Gene	Forward primer	Reverse primer	Gene ID/Reference
Ccl2	5'- *CAGGTGTCCCAAAGAAGCTG* -3'	5'- *CTCATTTGGTTCCGATCCAG* -3'	20296
Ccl3	5'- *GCAACCAAGTCTTCTAGCG* -3'	5'- *AGCAAAGGCTGCTGGTTTCA-* -3'	20302 / [S1]
Cxcl2	5'- *CCCTGGTTCAGAAAATCATCC* -3'	5'- *CTTTGGTTCTTCCGTTGAGG* -3'	20310
Cxcl3	5'- *TCATCAAGAAGATACTGAAGAGCG* -3'	5'- *AAAGACACATCCAGACACCG* -3'	330122
Cxcl5	5'- *TGGATCCAGAAGCTCCTGTG* -3'	5'- *CTTCACTGGGGTCAGAGTCC* -3'	20311
Cxcl10	5'- *CGTCATTTTCTGCCTCATCC* -3'	5'- *ATGGCCCTCATTCTCACTGG* -3'	15945
Cxcl11	5'- *AAGCTCGCCTCATAATGCAG* -3'	5'- ACAGCAGAGGGTCAGGTTCC -3'	56066
Cxcl12	5'- *CTCCAAACTGTGCCCTTCAG* -3'	5'- *CTACTGGAAAGTCCTTTGGG* -3'	20315
Hdac7	5'- *TGACCTCACGGCCATCTGTG* -3'	5'- *GGTTGGGTTTCTGTTTCCAG* -3'	56233
Ifng	5'- *TCAAGTGGCATAGATGTGGAAGAA* -3'	5'- *TGGCTCTGCAGGATTTTCATG* -3'	15978 / [S2]
Il1b	5'- *ACAAGGAGAACCAAGCAACG* -3'	5'- *TTGGGTATTGCTTGGGATCC* -3'	16176
Il2	5'- *TTGTGCTCCTTGTCAACAGC* -3'	5'- *CTGGGGAGTTTCAGGTTCCT* -3'	16183 / [S2]
Il6	5'- *CTCTGGGAAATCGTGGAAAT* -3'	5'- *CCAGTTTGGTAGCATCCATC* -3'	16193 / [S2]
Il10	5'- *ATAACTGCACCCACTTCCCA* -3'	5'- *GGGCATCACTTCTACCAGGT* -3'	16153 / [S2]
Rplp2	5'- *TACTAGACAGCGTGGGCATC* -3'	5'- *CAACACCCTGAGCGATGACA* -3'	67186 / [S2]
Tnfa	5'- *TCTCAGCCTCTTCTCATTCC* -3'	5'- *GCCATAGAACTGATGAGAGG* -3'	21926 / [S3]
Tlr4	5'- *CTTCATTCAAGACCAAGCCTTTC* -3'	5'- *AACCGATGGACGTGTAAACCAG* -3'	21898

References

S1. Hohjoh, H.; Horikawa, I.; Nakagawa, K.; Segi-Nishida, E.; Hasegawa, H. Induced mRNA expression of matrix metalloproteinases *Mmp-3*, *Mmp-12*, and *Mmp-13* in the infarct cerebral cortex of photothrombosis model mice. *Neurosci. Lett.* **2020**, 739, 135406. Doi: 10.1016/j.neulet.2020.135406.

S2. Razali, N.; Hohjoh, H.; Inazumi, T.; Maharjan, B.D.; Nakagawa, K.; Konishi, M.; Sugimoto, Y.; Hasegawa, H. Induced prostanoid synthesis regulates the balance between Th1- and Th2-producing inflammatory cytokines in the thymus of di-et-restricted mice. *Biol. Pharm. Bull.* **2020**, 43, 649–662. Doi: 10.1248/bpb.b19-00838.

S3. Razali, N.; Horikawa, I.; Hohjoh, H.; Yoshikawa, C.; Hasegawa, H. Prostaglandin-modulated interaction of thymic progenitor cells with blood vessels during estradiol-induced thymic involution. *BPB Rep.* **2019**, 2, 39–47. Doi: 10.1248/bpbreports.2.4_39.
